# A Provisional Gene Regulatory Atlas for Mouse Heart Development

**DOI:** 10.1371/journal.pone.0083364

**Published:** 2014-01-08

**Authors:** Hailin Chen, Vincent VanBuren

**Affiliations:** Department of Medical Physiology, Texas A&M HSC College of Medicine, Temple, Texas, United States of America; Harbin Institute of Technology, China

## Abstract

Congenital Heart Disease (CHD) is one of the most common birth defects. Elucidating the molecular mechanisms underlying normal cardiac development is an important step towards early identification of abnormalities during the developmental program and towards the creation of early intervention strategies. We developed a novel computational strategy for leveraging high-content data sets, including a large selection of microarray data associated with mouse cardiac development, mouse genome sequence, ChIP-seq data of selected mouse transcription factors and Y2H data of mouse protein-protein interactions, to infer the active transcriptional regulatory network of mouse cardiac development. We identified phase-specific expression activity for 765 overlapping gene co-expression modules that were defined for obtained cardiac lineage microarray data. For each co-expression module, we identified the phase of cardiac development where gene expression for that module was higher than other phases. Co-expression modules were found to be consistent with biological pathway knowledge in Wikipathways, and met expectations for enrichment of pathways involved in heart lineage development. Over 359,000 transcription factor-target relationships were inferred by analyzing the promoter sequences within each gene module for overrepresentation against the JASPAR database of Transcription Factor Binding Site (TFBS) motifs. The provisional regulatory network will provide a framework of studying the genetic basis of CHD.

## Introduction

Congenital heart disease (CHD) has been reported to occur in 5 to 8 per 1,000 live births [1]. Due to its pathological severity, CHD has become an important public health issue as a leading cause of infant mortality. According to a clinical report on the infant mortality resulting from CHD in the US [2], [3], up until 2006, the infant mortality rate due to CHD decreased but was still as high as 37.69 per 100,000 live births. In addition to the high infant mortality rate, up to half of the surviving children will have the impaired neurodevelopmental outcomes across a wide spectrum of domains [1]. Other than cases of syndromic CHD, It is also believed that for fetuses with CHD, hypoxemia from the intracardiac mixing of blood can cause cerebral hypoxia, and inadequate fetal cerebral oxygen delivery thus results in impaired cerebral development [1]. It is thus crucial to understand the mechanisms of cardiac development to be able to elucidate the mechanisms of pathogenesis in CHD for improving diagnostic approaches and therapeutic strategies.

**Figure 1:**
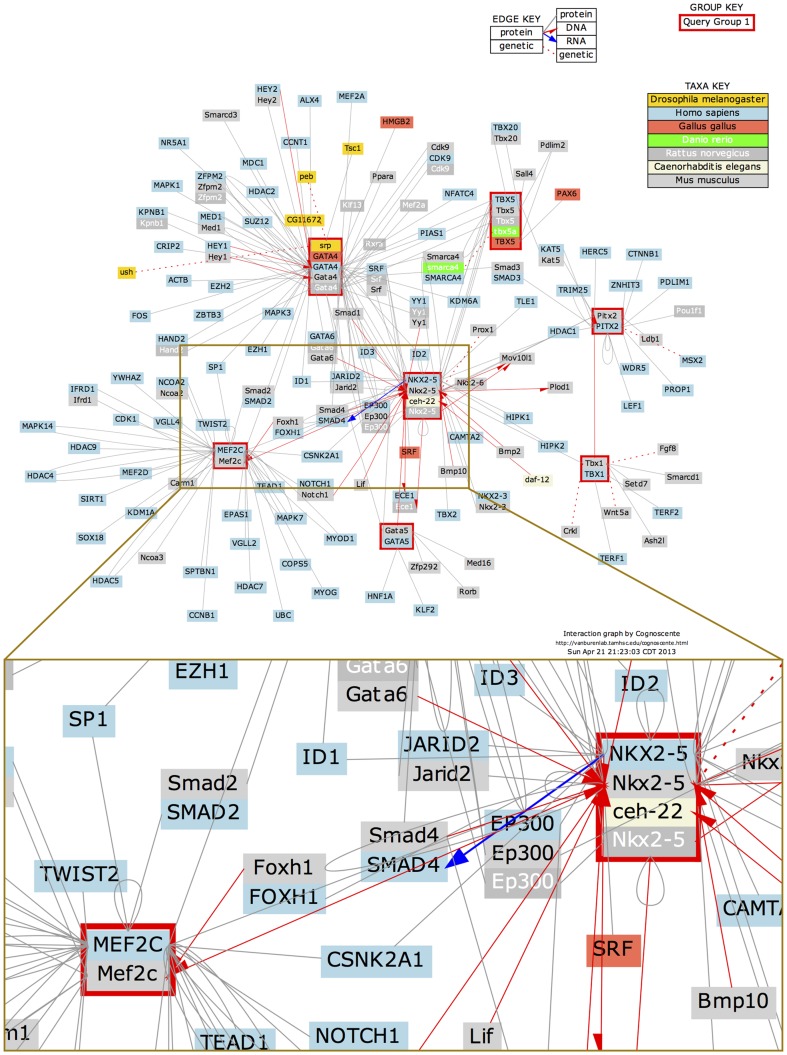
Molecular interaction network results from the literature-based Cognoscente knowledgebase where several genes with important roles in cardiac development were queried: Nkx2-5, Gata4, Gata5, Tbx1, Tbx5, Pitx2 and MEF2c (red boxes). Red arrows point from the TFs (proteins) to their targets (DNA), black solid lines represent protein-protein interactions and red dashed lines represent genetic interactions. Stacked boxes (which sometimes repeat the same gene name) in the figure show orthologs of genes in organisms where the ortholog has a documented interaction. Node colors indicate different model organisms as defined in the Taxa Key. Here, one example of the complicated transcriptional regulation is that Foxh1 transcription factor physically and functionally interacts with Nkx2-5 transcription factor to regulate the expression of Mef2c gene (inset region).

Several recent studies have focused on cardiac developmental genetics with the aim of identifying the genetic basis of congenital heart disease [4] (**Table S1**)[**5**]. Multiple transcription factor genes with defined functions in mouse cardiac development have been identified [6] (**Table 1**). To complement knowledge of the required normal gene functions during cardiac development, investigators are digging deeper into the mechanisms corresponding to these gene functions by uncovering the molecular interactions that define developmental pathways. Some of the most important interactions to consider include protein-DNA interactions, protein-protein interactions and genetic interactions (genetic associations without a clearly defined molecular mechanism). **Figure 1** gives an example of a molecular interaction network comprising several genes that are important in mouse cardiac development. This interaction network graph was generated using Cognoscente, which provides a knowledge base of biomolecular interactions supported by the literature [7]. **Figure 1** illustrates an example of complex transcriptional regulation during mouse cardiac development, where the Foxh1 transcription factor physically and functionally interacts with the Nkx2-5 transcription factor to combinatorially regulate transcription of the Mef2c gene, while the Mef2c protein product is also a known transcription factor that directly regulates the transcription of the Calreticulin gene and the BOP gene (not shown in this graph) [8]–[10]. Therefore, for a comprehensive understanding of cardiac development, elucidation of the transcriptional signal propagation between transcription factors (TFs) or between TFs and their non-TF targets in the genomic scope is one of the highest priorities (see examples of the transcriptional signal propagation in **Figure S1**, which summarizes the literature-based mouse transcriptional interactions from the public version of the TRANSFAC database [11], [12]). This knowledge is conveyed in the structure of a Transcriptional Regulatory Network (TRN). A TRN is the collection of connected transcriptional interactions involved in transcription-level regulation of a developmental process, homeostatic process, or disease process. Representations of TRNs by Cognoscente contain hypernodes which may correspond to genes or their corresponding products (e.g. protein), while edges with a red arrow pointing from the TF to its target gene represent a physical interaction between the TF protein and their target genes. One possible role for protein-protein interactions is combinatorial regulation by TFs of a specific target. Deciphering the details of underlying TRNs is a major goal of modern research in cardiac development, and will result in an important resource for genetic counseling and for developing more effective treatment plans for congenital heart disease.

**Table 1:** Signature genes expressed in mouse during cardiac development.

Cardiac Crescent	Linear Heart	Chamber Formation	Maturation/Septation
Gata4	Gata4	Gata4	Gata4
Nkx2-5	Gata5	Nkx2-5	Nkx2-5
Mesp1/2		Tbx5	Tbx5
		dHand	RxRa
		eHand	FOG-2
		Pitx2	Pitx2
		MEF2C	Sox4
			NF-Atc
			TEF-1
			Tbx1
			Hey2
			CITED
			ZIC3

Cardiac development follows a procession of the following four stages: cardiac crescent, linear heart, chamber formation and finally maturation/septation. Gene functions during cardiac development are generally described in terms of these four milestones [5].

Sea urchin and Drosophila have been two of the most useful models for studying TRNs [13], [14]. Investigators have traditionally defined developmental TRNs by assembling knowledge of transcriptional regulation member molecules from individual experiments into a network structure. This laborious approach to elucidating TRNs from respective experiments of single (or several) transcriptional regulation(s) produces reliable biological information. However, elucidating the complete networks in more complex organisms, such as human or mouse, would be extremely difficult using this strategy, as much time and labor are required to characterize the role of just one gene in a physiological or pathological state. The strategy described above is the bottom-up approach of network construction. Computational strategies offer a top-down approach to network construction in the genome-wide scope that complements the bottom-up approach. In the wake of biotechnology advancements, high-content experimental data is fueling this top-down research to foster TRN reconstruction in the genome-wide scope using computational strategies. Previous studies have applied the top-down approach on high-content experimental data to examine the cardiac TRN comprised of several key TFs [15], and to infer spatio-temporal protein networks active in human heart development [16].

There are several popular computational strategies for inferring TRNs, including Boolean networks, Bayesian networks, systems of differential equations, genome-wide pairwise correlation and genome-wide pairwise Mutual Information [17]. However, each of them has certain drawbacks. The Boolean algorithm assigns each variable a binomial value, which could omit important information about multinomial/continuous variables. Bayesian network construction is very promising for representing and inferring causal relationships, but this strategy is only effective for the construction of small TRNs, due to the super-exponential increase in algorithm running time for large networks. Defining a differential equation model of a TRN requires knowing the equation of dynamics, then calculating the parameters to optimize the TRN model against real data. However, deriving an appropriate equation of dynamics remains as a challenge. Furthermore, solving a differential equation system of any realistic complexity presents a challenge. As to the correlation and mutual information algorithms, manually setting the appropriate thresholds without a principled reference poses difficulties in most cases. Strategies applying algorithms with these drawbacks are thus not satisfying. In recent decades, a large amount of experimental information about biological networks has been collected, and opportunities have thus increased for deciphering their topological features, including defining their identity as scale-free networks, small world networks, adaptive motifs, feed-back motifs, ‘AND’ and ‘OR’ logic motifs and modular networks (Klipp, E. et al. 2009). This motivates a systematic effort of determining network topological features, which will benefit the effectiveness and utility of network reconstruction. High modularity is one of the most accepted network topological features of TRNs [18], [19]. Modularity is a measure of the structure of networks, where networks with high modularity have dense connectivity between nodes within defined modules (or groups), and relatively sparser connectivity between nodes from different modules.

Based on the modularity feature of TRNs, we first describe a novel computational strategy for inferring the TRN of mouse cardiac development in the genomic scope. We developed an approach of comprehensive clustering to retrieve the optimal set of co-expression gene modules by analyzing the transcriptomic data associated with mouse cardiac development. We defined the optimal set of modules using the Davis-Bouldin Index (see RESULTS). We then identified modules with phase specific expression activity in the phases of mouse cardiac development. Next we applied an alignment strategy that identifies the putative transcription factor binding sites (TFBS) along gene promoter sequences of every selected module, and determined overrepresented TFBS(s) whose corresponding TF(s) may initialize co-expression in the module via co-regulation. Overrepresented pathways represented in each module were also annotated. We created a graphic representation of inferred transcriptional regulations to visualize the provisional reconstruction of the TRN for mouse cardiac development (The transcriptional regulations were represented in the graph as TFs with arrows pointing to their corresponding targets). Finally, we applied the source data of sampled TF genomic binding sites from the ENCODE database to evaluate the provisional transcriptional regulations from our analysis. Taken together, we have established the novel computational strategy for inferring the TRN in mouse cardiac development that advances a provisional gene regulatory atlas.

## Results

### Retrieving the optimal set of co-expression gene modules corresponding to the minimal Davies-Bouldin Index

Modularity provides a control structure for silencing or activating discrete parts of a network. Modularity of a biological network thus provides a selection advantage that appears to have been conserved through evolution [20]. Modularity of TRNs follows this tendency to be well conserved in the complex organisms, such as human and mouse [21]–[24]. In biological networks, modules can be understood in terms of subsystems. The modularity of TRNs can be represented by co-expression gene modules inside each of which the modular genes are expressed covariantly across a large collection of expression sets of different samples [18], [19]. Here we leveraged mouse as a model to study the TRN of cardiac development with our computational strategy based on the modularity feature of co-expression. We used publicly available microarray data from the Gene Expression Ominbus (GEO), from which we collected transcriptomic data from 239 selected microarray experiments (arrays) as our source data (see MATERIALS AND METHODS). These experiments are all based on mouse heart tissues or mouse embryonic stem cells (i.e. heart lineage samples).

**Figure 2:**
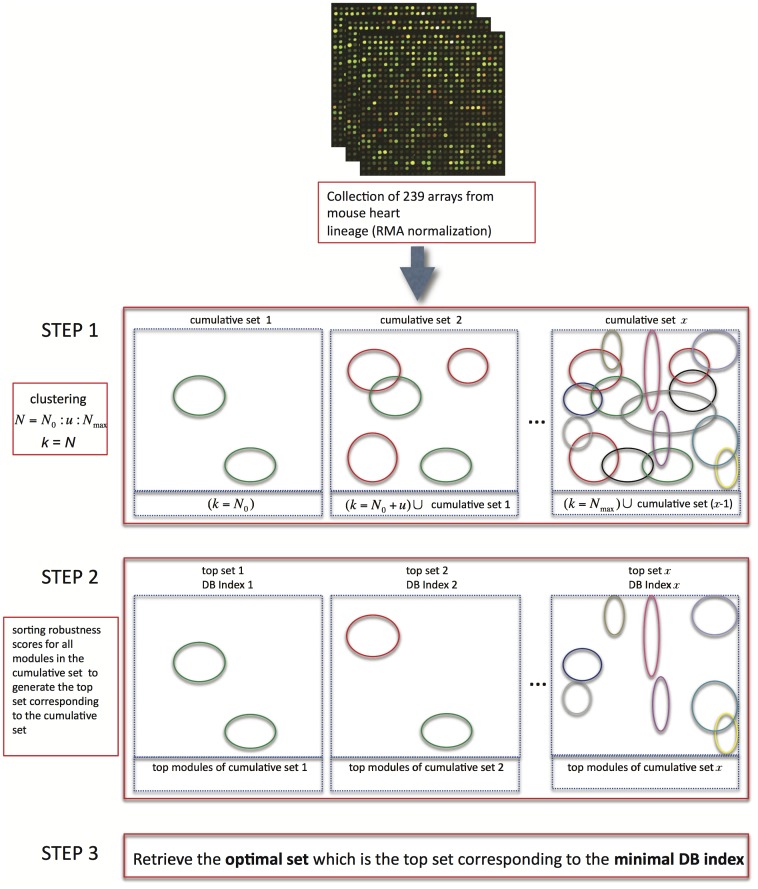
Computational strategy of our comprehensive clustering approach. 239 selected arrays for distinct phases of mouse cardiac development were used as source transcriptomic data. In step 1, for each iteration (N = N_0_∶u∶N_max_) a cumulative set of modules is constructed using k-means clustering of the current iteration (*k* = *N*, circle represents a module, where circles of the same color are from one instance of k-means clustering) and this instance is combined with the cumulative set of modules from the previous iteration (cumulative set 1, cumulative set 2, … cumulative set x). In step 2, the top set from each of the cumulative sets is created by selecting the modules corresponding to the top robustness scores to cover all the genes on the array (top set 1, top set 2, … top set x). The Davis-Bouldin Index (DB Index) of every top set is calculated to measure modular configuration in the set (DB Index 1, DB Index 2, … DB Index x). In step 3, the minimal calculated DB Index describes the best modular configuration and corresponds to the optimal set of co-expression gene modules. (see details in METHODS).

By leveraging the modularity feature of TRNs, we have the opportunity to infer TRNs when the co-expression gene modules are properly retrieved. Module structure implies the coordination of TFs responsible for the co-expression in such modules. Clustering, a type of unsupervised machine learning, is the routine approach used to identify co-expression gene modules from transcriptomic data [25]. However, due to the non-overlapping design of standard clustering algorithms, each gene is assigned membership to only one specific co-expression gene module. This is not likely to accurately represent biological circuits, where a gene may have multiple TF regulatory binding sites on the promoter and may thus be the member of multiple co-expression gene modules [18]. Moreover, modular overlap has been revealed in many biological network systems [26], [27]. Therefore, some genes have multiple modular memberships of co-expression in the TRN due to the complexity of TF regulation. There are thus compositional overlaps in the membership of biological modules. We designed a novel comprehensive clustering algorithm (**Figure 2**; see MATERIALS AND METHODS) as an improvement to non-overlapping clustering strategies to retrieve overlapping co-expression gene modules. Systematic optimization was used in this clustering approach for selecting the most likely coregulated genes from several sets of co-expression gene modules. We defined the optimal set of co-expression gene modules as the set of modules that obtained the best configuration of those modules in the genomic scope as evaluated by the Davis-Bouldin Index (DB Index). This best configuration of modules is a best approximation of biological co-expression gene modules given the selected data [18], [19].

**Figure 3:**
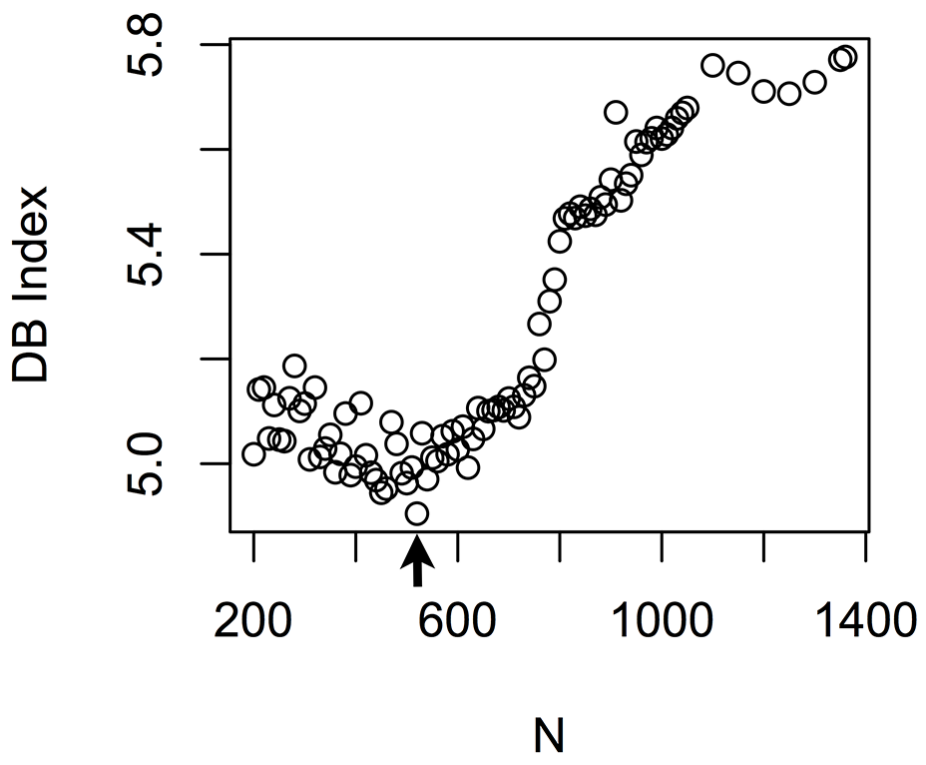
DB Index as a function of N. N is the number of iterations of comprehensive clustering used specify each top set of co-expression gene modules. The arrow marks the lowest calculated DB Index of 4.9, where N = 520. Lower DB Index values correspond to better configuration, so the top set of clusters for N = 520 is the optimal set of co-expression gene modules.

We applied this strategy of comprehensive clustering to generate a plot of DB Indices throughout the computed top sets (**Figure 3**) of co-expression gene modules. The DB Index for the top set where N = 520 was the minimum computed DB Index (4.9047), which corresponds to the best configuration of modules. We found around 34.6% genes have more than one modular membership within the set. The optimal set of 765 co-expression gene modules that we computed for the mouse cardiac development dataset are listed in **Table S2**. All 15711 selected gene features were included in the 765 co-expression gene modules. The maximal re-occurrence of a gene in different modules is 8, and the mean occurrence of genes in different modules is 1.49. We compared this improved clustering algorithm with other overlapping clustering algorithms: fuzzy clustering and biclustering. We set the cluster number to 765 for the other clustering algorithms to obtain respective sets of co-expression gene modules from the cardiac development microarray sets. We applied gene ontology term enrichment tests (molecular function category only) to identify the enriched molecular functions in each set where the ratio of modules having at least one term enriched out of all the modules is the metric used to make the comparison [28]. We found the improved clustering (272/765) and fuzzy clustering (271/765) shared similar degree of annotated modules in their respective sets, while biclustering resulted with very few modules when we set the parameter related to cluster number to 765 and none of them was annotated by any enriched term. Therefore, we can conclude that our improved clustering outperforms biclustering, and it performs as good as fuzzy clustering when parameters for fuzzy clustering are chosen appropriately (e.g. fuzziness and memberships for each observation/gene).

### Phase specific expression activity in inferred modules for mouse cardiac development

We analyzed expression profiles for the co-expression gene modules during the period of prenatal cardiac development (E10.5∼E18.5) using the arrays in the dataset that were associated with these distinct phases of development (GSE1479). In order to inspect whether there is differential expression among the phases in the modules, we first grouped the arrays based on the seven phases: E10.5, E11.5, E12.5, E13.5, E14.5, E16.5 and E18.5. We then graphed the expression trace of each module in the developmental time order of the seven phases. For each module, we compared the expression among phases to identify the phase specific expression within the module.

**Figure 4:**
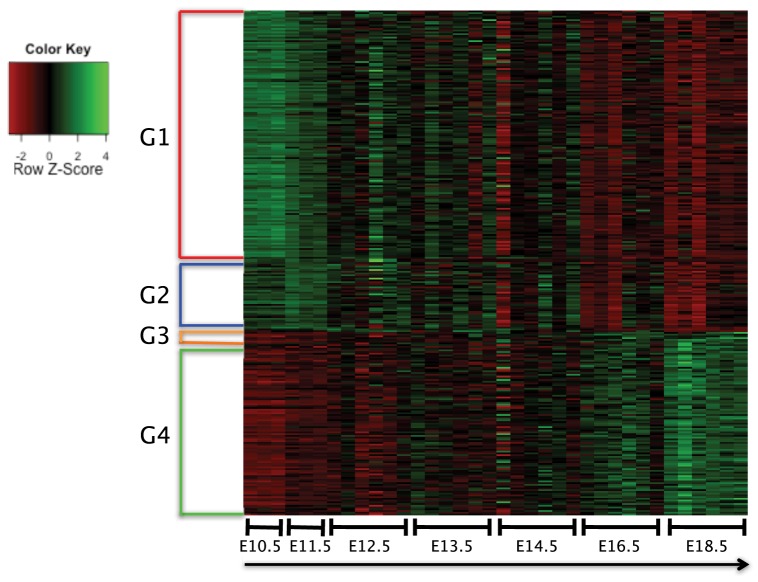
Heatmap of expression profiles across the cardiac developmental phases (E10.5–E18.5) for sampled modules. The modules in group 1 (G1) have the highest level of expression at E10.5; the modules in group 2 (G2) have the highest level of expression at E11.5; the modules in group 3 (G3) have the highest level of expression at E16.5; the modules in group 4 (G4) have the highest level of expression at E18.5.

**Figure 4** shows differential expression in phases for sampled modules. Modules in group 1 have the highest level of expression at E10.5; modules in group 2 have the highest level of expression at E11.5; modules in group 3 have the largest level of expression at E16.5 and the modules in group 4 have the highest level of expression at E18.5. We analyzed the whole optimal set of modules and found that (1) 55.4% modules have significantly different expression (FDR adjusted p<0.05) among the phases; (2) 46% modules have significantly different expression (FDR adjusted p<0.05) between E10.5 and the other phases; (3) 33.2% modules have significantly different ial expression (FDR adjusted p<0.05) between E11.5 and the other phases; (4) 16.1% modules have significantly different expression (FDR adjusted p<0.05) between E16.5 and the other phases; (5) 35.9% modules have significantly different expression (FDR adjusted p<0.05) between E18.5 and the other phases (**Table S3**). We assigned each of the modules to pattern groups corresponding to their phase specific expression.

**Figure 5:**
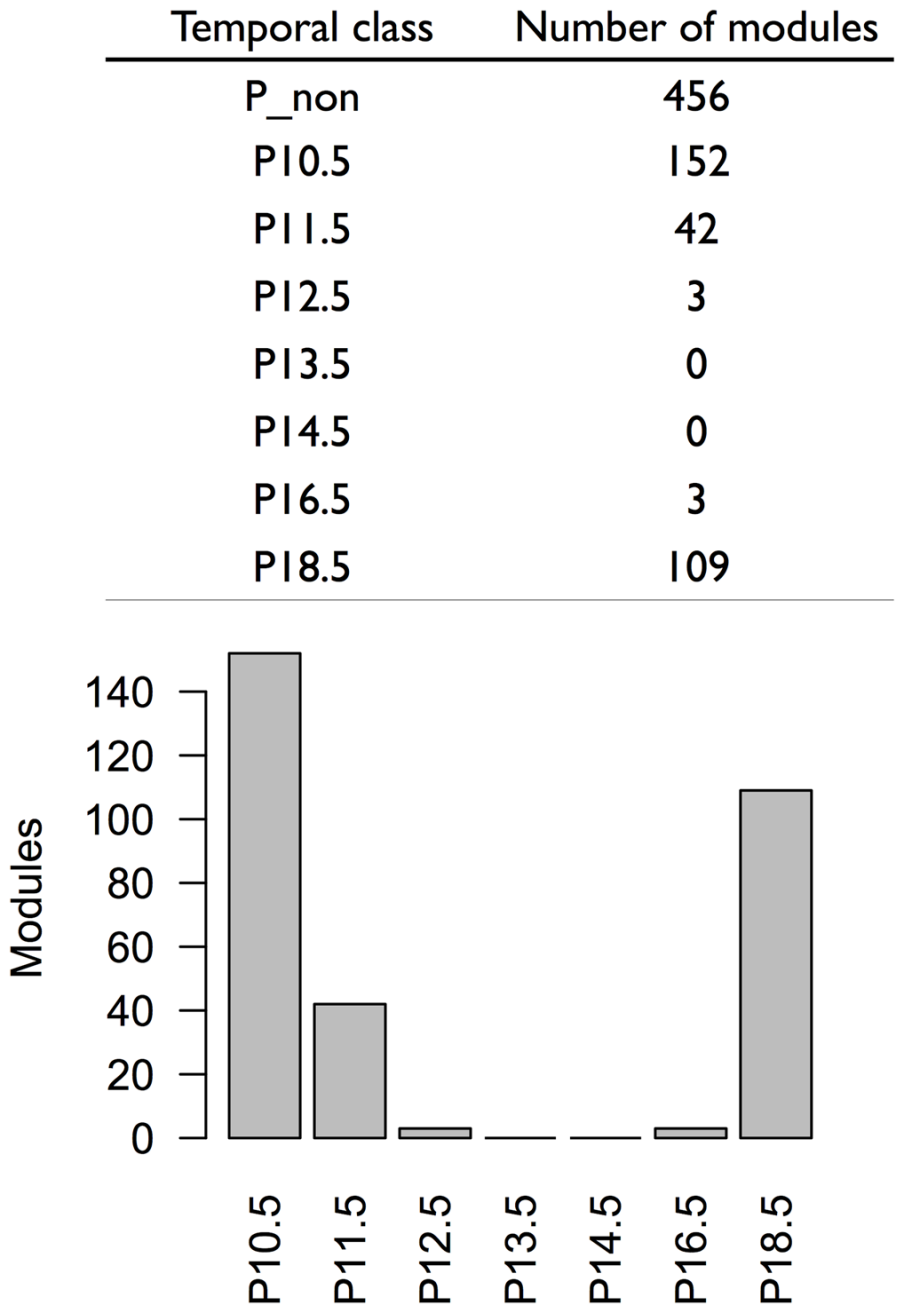
The optimal module set was classified into eight categories according to the phase specific expression in the modules. The table and bar plots summarize the classifications.

Module phase categories were assigned in the table of differential expression analysis (**Table S3**) and module assignments were summarized according to the phase specificity of gene expression in the modules (**Figure 5**): category P_non (includes modules having no significantly different expression among phases), category P10.5 (includes modules having the significantly largest level of expression at E10.5), category P11.5 (includes modules having their significantly highest level of expression at E11.5), category P12.5 (including modules having their significantly highest level of expression at E12.5), category P13.5 (includes modules having their significantly highest level of expression at E13.5), category P14.5 (includes modules having their significantly highest level of expression at E14.5), category P16.5 (includes modules having their significantly highest level of expression at E16.5) and category P18.5 (includes modules having the ir significantly highest level of expression at E18.5).

### Known molecular pathways formalize the developmental program in mouse heart

**Figure 6:**
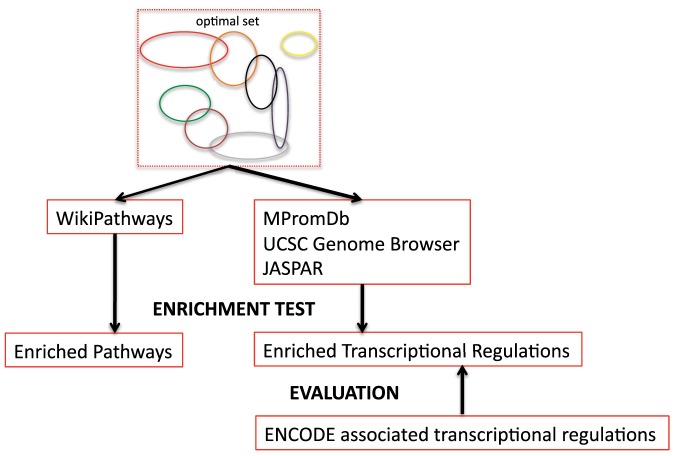
Flow chart summarizing further analysis after computing the optimal set of co-expression gene modules. We applied the hypergeometric enrichment test to each module to inspect the overrepresentation of known pathways central to mouse cardiac development. In parallel, hypergeometric enrichment tests were performed for transcription factor binding sites (TFBS) in modules to obtain the overrepresented/enriched TFBSs and infer the corresponding transcriptional regulations comprising the TRN in the mouse cardiac developmental program. Finally, the ENCODE associated transcriptional regulations were used as a reference set to test the performance of our strategy for inferring the TRN.

The symphony of life is performed by numerous molecular reactions in an interconnected network. The great abundance of new high-content experimental data is fueling an understanding of molecular pathways with the formalized composition of interconnecting reactions. Wikipathways provides an open and collaborative platform dedicated to the curation of experimentally-supported biological pathways [29], [30]. In order to inspect the known molecular pathways implementation in the developmental program of mouse heart, we developed a strategy leveraging the Wikipathways knowledgebase to estimate the overrepresentation of known mouse molecular pathways throughout our optimal set of co-expression gene modules (**Figure 6**; see MATERIALS AND METHODS).

We obtained 161 well-known mouse molecular pathways from the Wikipathways knowledgebase. For each module from the optimal set for mouse cardiac development, we applied enrichment tests to inspect the overrepresentation of the known pathways in the module. We thus obtained a table of pathway overrepresentations (FDR adjusted p<0.05) from systematic enrichment tests (**Table S4**). Overrepresented pathways may have central roles in the developmental program of mouse heart.

**Figure 7:**
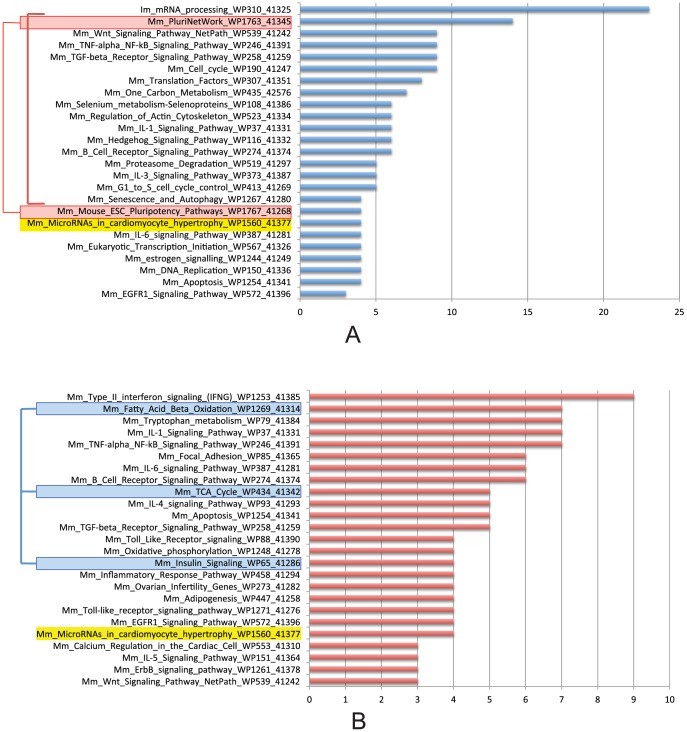
Overrepresented pathways for P10.5 and P18.5. A. The top 25 overrepresented pathways in P10.5, where modules in this class have their highest level of expression at E10.5. Pathways in red boxes are pluripotency-related pathways enriched in P10.5 modules; pathways highlighted in yellow are also enriched in P18.5. B. The top 25 overrepresented pathways in P18.5, where modules in this class have their highest level of expression at E18.5. Pathways in blue boxes are metabolism-related pathways enriched in P18.5 modules; pathways highlighted in yellow are also enriched in P10.5.

As every module was assigned to one phase category according to the highest level of modular expression in that phase, the overrepresented pathways in a category are highly expressed in the corresponding phase. **Figure 7** shows the most frequently overrepresented pathways (top 25) in P10.5 (A) and P18.5 (B), where module members are highly expressed at E10.5 and E18.5, respectively. In the phase of E10.5, pluripotency-related pathways are highly expressed; while in the phase of E18.5, metabolism-related pathways are highly expressed. The two featured series of pathways fit well with their respective phases of early development and late development in mouse heart. In addition, we found the Wnt, TGF-beta and EGFR1 signaling pathways were highly expressed in the phase of E10.5. The interferon, TNF-alpha, NF-kB, B cell receptor, inflammatory response, Toll-like receptor, IL1, IL4, IL5 and IL6 signaling pathways are highly expressed in the phase of E18.5. We also found that the two phases shared one highly expressed pathway: MicroRNAs in cardiomyocyte hypertrophy. These pathways are highly expressed to mediate the respective phase transitions of mouse cardiac development.

### Transcriptional regulations driving the developmental program of mouse heart

JASPAR is a curated database that collects known Transcription Factor Binding Site (TFBS) motifs in various organisms from the experiment-based literature [31]. We selected mouse-specific TFBS motifs and mouse-homologous TFBS motifs as the baits in a promoter pool (promoters were obtained from MPromDb and the UCSC Genome Browser) of every co-expression gene module from the optimal set for mouse cardiac development to identify possible TFBSs on the modular gene promoters via sequence alignment tests. Once we identified the possible TFBSs, we inferred the possible transcriptional regulations between the corresponding TFs and the module. However, alignments can arise from random sequence variation that produces false positive hits of TFBS motifs on the gene promoters. We therefore applied TFBS enrichment tests to each module to exclude the false-positive cases in our prediction of transcriptional regulations for each module (**Figure 6**, see details in MATERIALS AND METHODS).

We obtained 160 known TFBS motifs (mouse-specific and mouse-homologous) from JASPAR. For each module from the optimal set for mouse cardiac development, we applied enrichment tests to inspect the overrepresentation of the known TFBS motifs in this module. We compiled a table of TFBS motif overrepresentation (FDR adjusted p<0.05) from these systematic enrichment tests to infer the putative transcriptional regulations of the modules (**Table S5**). The predicted transcriptional regulations are the basic components of our provisional TRN of mouse cardiac development.

**Figure 8:**
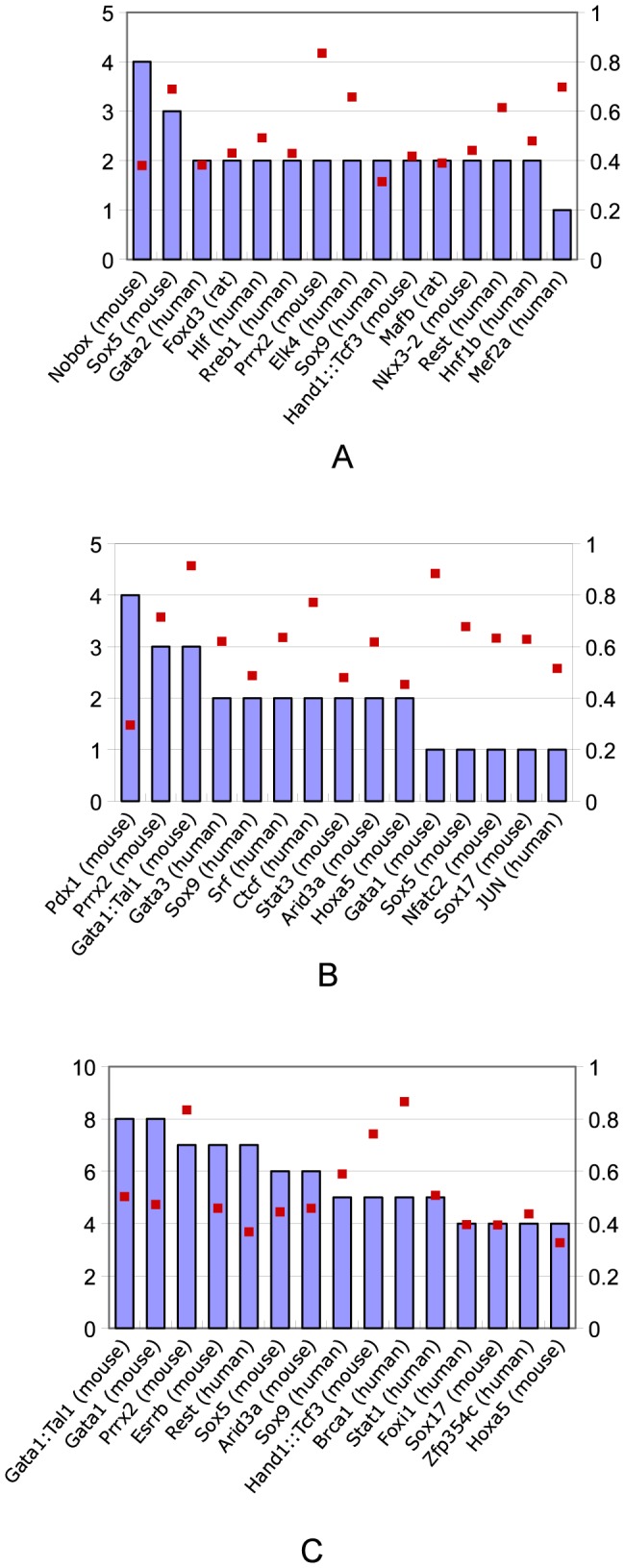
The top 15 TFs (mouse, human or rat) corresponding to the most frequently overrepresented TFBS motifs having a Pearson correlation > = 0.3 with module expression in phase categories. P10.5 (A), P11.5 (B) and P18.5 (C). Blue bars represent frequency of TFBS motif overrepresentation (left axis); red dots show the Pearson correlation of expression (right axis).

TFs corresponding to the overrepresented TFBS motifs in a category are likely to be responsible for the increased expression in the corresponding phase. **Figure 8** shows the TFs that correspond to the most frequently overrepresented TFBS motifs (top 15 from mouse, human and rat) that have a Pearson correlation > = 0.3 with module expression in categories: P10.5 (A), P11.5 (B) and P18.5 (C). Expression of modules in these phases is thus likely mediated by TFs for the respective overrepresented TFBS. In the phases of E10.5, E11.5 and E18.5, which denote the early and late heart development, several TFs such as Sox5, Sox9 and Prrx2, are shared as the top-ranked lists of TFs across these developmental phases. There are also TFs in the list of top-ranking overrepresented TFBSs that are uniquely enriched in distinct phases. For example, Gata2, Nkx3-2 and Mef2a are enriched in E10.5 modules, Gata3, Stat3 and Jun in of E11.5 modules, Brca1 and Stat1 in E18.5 modules. Taken together, TFs work together both across phases and in a phase-specific manner to regulate phase specific expression and mediate phase transitions during normal cardiac development.

We integrated the table of pathway overrepresentation with the table of TFBS motif overrepresentation to assign the TFs corresponding to those TFBS motifs with pathway annotations, thus providing clues for TF involvement in known molecular signaling pathways for mouse cardiac development (**Table S6**).

### A provisional transcriptional regulatory atlas of mouse cardiac development

**Figure 9:**
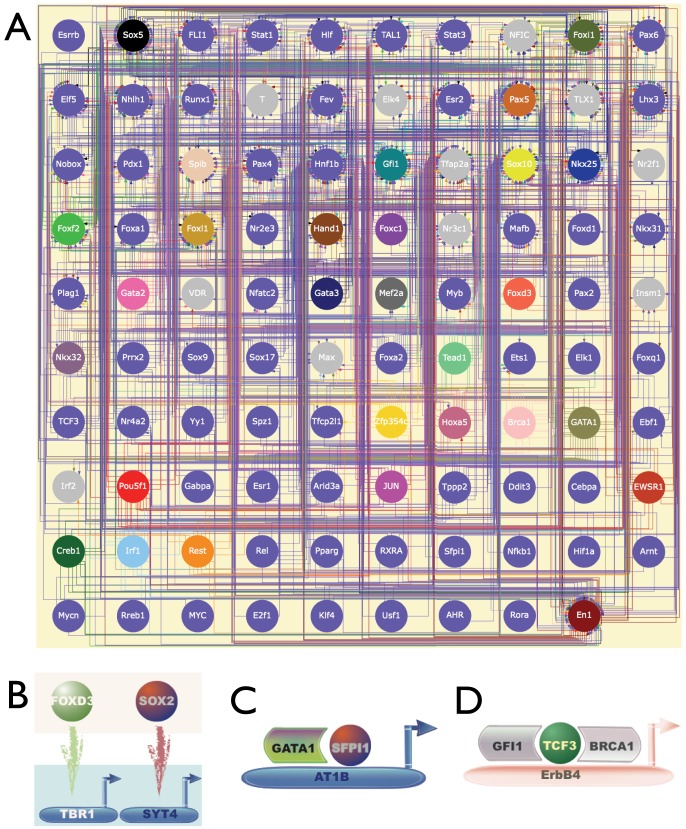
Partial visualization of the TRN for mouse cardiac development. A. Sub-network structure of the TRN, focusing on the sampled TFs only. Nodes and their respective outgoing arrows are colored to help clarify arrow paths. B. An explanatory example showing TFBS non-aligned transcriptional regulations. Foxd3 and Sox2 are in a co-expression gene module (beige box) and their individual TFBS aligned gene targets Tbr1 and Syt4 are in another co-expression gene module (light blue box). As shown, Syt4 is TFBS non-aligned gene target for Foxd3 and Tbr1 is the TFBS non-aligned gene target for Sox2, as Foxd3 and Sox2 are inferred TFs regulating Tbr1-Syt4 modular transcription. More complex scenarios for explaining co-regulation for non-aligned TFBS motifs are possible. C and D show combinatorial transcriptional regulations in the TRN inferred from the integration of the inferred transcriptional regulations with documented TF protein – TF protein interactions.

To develop a network structure conveying the transcriptional regulations of mouse cardiac development in the genome-wide scope, the TRN was represented using the Graphviz network drawing tool to present the transcriptional regulations inferred in the previous section. TF-gene regulations were established by assigning every inferred TF-module regulation to each gene member of the module. We distinguish between two types of TF-gene regulations: TFBS-aligned regulations where a specific TFBS is found on the regulated gene promoter and TFBS-non-aligned regulation where the specific TFBS is not found on the regulated gene promoter (**Table S7**). There are several possible explanations for the TFBS non-aligned regulations, including incomplete information of the TFBS motifs. We examined the inferred transcriptional regulations and identified a recurring pattern whereby multiple TFs in the same module regulate individual genes in another module. In these cases, target genes had similar expression profiles because their respective TF regulators had similar expression profiles (**Figure 9B**). In these examples, non-aligned targets may be thought of as indirect or incidental regulatory targets. Because of the evident likelihood that non-aligned targets were indirect targets, we only used TFBS-aligned regulations as transcriptional regulations in the assembly of the provisional TRN of mouse heart development. To summarize the provisional TRN, we assembled the core network of regulations between sampled mouse transcription factors (**Figure 9A**). As the core of the TRN, the transcriptional regulations among sampled TFs formed a sub-network where the number of edges is ten-fold greater than the number of nodes. This graph lifts a corner of the complexity in the TRN of mouse cardiac development.

**Figure 10:**
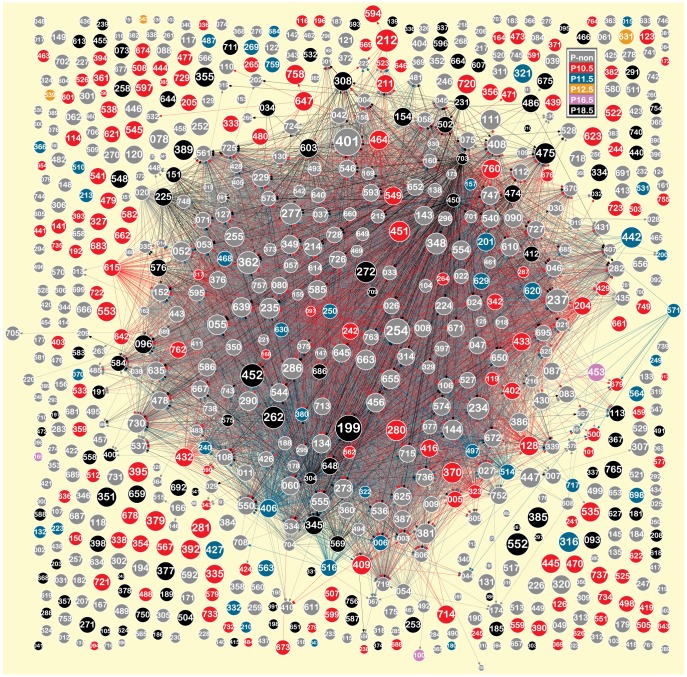
Provisional atlas of TRN modules for cardiac lineage development. Nodes are labeled with module index numbers. Node diameter is proportional to the log_10_ of the number of genes in each module, node color corresponds to phase class as shown in the legend, and directed edges (same color as the module they point from) represent transcriptional regulation between modules.

Transcriptional regulation of a target gene is the collaborative work among several TFs, co-activators and co-repressors. Therefore we integrated a compendium of documented knowledge of TF protein – TF protein interactions with the inferred transcriptional regulations to define the TRN of mouse cardiac development [32] **Table S8**: TF regulatory complex of 2 TFs; **Table S9**: TF regulatory complex of 3 TFs). These combinatorial regulations are well supported by evidence that the target gene is regulated by multiple interacting TFs. Gata1 and SFPI1 are well-studied examples having a physical interaction, and are found in our provisional TRN to co-regulate AT1B in a co-expression gene module that doesn't show differential expression across developmental phases (**Figure 9C**). Our result is consistent with AT1B being previously defined to play key role in mouse normal cardiac morphology [33]. GFI1, TCF3 and BRCA1are the well-studied in development and cancer. From the provisional TRN, we infer that these TFs co-regulate ErbB4 (**Figure 9D**). This novel complex regulation was retrieved in a co-expression gene module that has its significantly highest level of expression at E18.5. We note that not every documented TF – TF interaction was successfully incorporated with the inferred regulations to offer a complete description of combinatorial regulation of gene transcription. Coverage of the array platform used in the definition of gene modules is incomplete, and we posit that the context of heart lineage development is central to our findings, so we expect that gene regulations specific to other contexts will not be identified with the dataset employed here. To summarize the TRN at the modular scope, we graphed modules so that node diameter is proportional to the log_10_ of module size (number of genes in module), node color corresponded to phase category (as measured by phase specific expression), and directed edges were drawn between modules containing transcription factors and those modules containing their respective inferred targets (**Figure 10**).

### Evaluation of the provisional transcriptional regulatory network using the ENCODE database

The ENCODE database offers source data from large-scale ChIP-seq experiments of selected TFs to identify the corresponding DNA binding sites in mouse genome [34]. We obtained data of genomic binding sites for several TFs (Usf1, Tcf3, Tbp, Tal1, Srf, Pax5, Nrf2, Max, Gata2, Gata1, Ets1, Ctcf, Cmyc, Cjun) from the ENCODE database to evaluate the inferred transcriptional regulations of those TFs in our provisional TRN of mouse cardiac development. Based on the ChIP-seq data, the regulated gene pool of every sampled TF was estimated from the analysis for the location associations between its genomic binding sites and the gene promoters. The metric used in the evaluation is the ratio of the number of the inferred transcriptional regulations for a specific TF found in the regulated gene pool based on the ChIP-seq data for this TF (regulations in agreement) to the number of all the inferred transcriptional regulations (regulations inferred from enrichment tests) for this TF (**Table 2**).

**Table 2:** Summary of the agreements between the inferred transcriptional regulations and the regulations obtained from ChIP-seq data in the ENCODE database for several sampled TFs.

TF	Regulations in agreement	Regulations inferred from enrichment tests	Ratio
Usf1	38	220	0.17
Tcf3	160	2867	0.06
Tbp	1492	3807	0.39
Tal1	78	126	0.62
Srf	0	2	0
Pax5	0	127	0
Nrf2	0	22	0
Max	5	32	0.16
Gata2	419	3736	0.11
Gata1	3049	4102	0.74
Ets1	289	467	0.62
Ctcf	7	8	0.88
Cmyc	40	132	0.30
Cjun	973	3041	0.32

This ratio varies across the sampled TFs. ENCODE projects were performed on specific cell lines and for specific experimental conditions. Unfortunately, relatively few ENCODE samples are relevant to cardiac development. Access to TF binding sites on the genome is dynamic across diverse cell or tissue types [35]. Therefore, computing this ratio gives a sense of the concordance of ENCODE results and our inferences, but is not able to completely evaluate the inferred transcriptional regulations of the sampled TFs in cardiac development. However, the agreements between the predicted regulations in the TRN and the regulations obtained from ChIP-seq data in the ENCODE database support the analytical power of our novel algorithm for inferring several transcriptional regulations that were also inferred by another completely different analysis. We also show that ChIP-seq data for different cell types or different conditions have reasonably good agreement. Therefore, by obtaining additional ChIP-seq data of a sampled TF for heart lineage samples in the future, we expect that the ratio may become a more reliable metric to evaluate inferences.

## Discussion

Our novel algorithm to retrieve the optimal set of co-expression gene modules in the genomic scope from a large selection of microarray data associated with mouse cardiac development allows overlap among the modules to maximally approximate biological co-expression gene modules. Differential expression among phases in the modules was identified. We applied the hypergeometric enrichment test to identify overrepresented pathways of the modules to infer important pathways involved in the developmental program of mouse heart. These pathways thus provide important clues for understanding the mechanisms underlying the phase transitions during cardiac development. In order to infer the provisional TRN of mouse cardiac development, we applied the hypergeometric enrichment test to find true-positive transcriptional regulations for each module by identifying overrepresented TFBSs in the module. We further integrated documented TF protein – TF protein interactions with the inferred transcriptional regulations to include TF combinatorial regulation in the TRN of mouse cardiac development. Gata1 and SFPI1 were found in the TRN to co-regulate AT1B. GFI1, TCF3 and BRCA1 were found to co-regulate ErbB4. In addition, we used the obtained ChIP-seq data of genomic binding sites from the ENCODE database for the sampled TFs to evaluate the inference of the TRN. The agreements between the inferred regulations in the TRN and the regulations obtained from ChIP-seq data imply the inferential power of the novel algorithm.

With the fast development of the next generation sequencing (NGS), RNA-seq may become the mainstream technology for generating transcriptomic data based on improved accuracy and the capacity of detecting novel transcripts. Using better source transcriptomic data in our algorithm will infer the TRN with closer approximation to the biological TRN. DNA-seq is developing fast with the evolution of NGS. The genomic binding sites of sampled TFs from DNA-seq can be integrated with transcriptomic data by our algorithm to infer transcriptional regulations. However, due to the unavailability of molecular probes for many TFs, it will remain a challenge to infer TRNs at the genomic scope. In addition to the genetic regulation, epigenetic regulation is an important contributor to transcriptional regulation [36]. One future direction for this work is to integrate the inferred TRN with known epigenetic regulations to define important connections between genetic and epigenetic regulation in cardiac development.

A disease is rarely a consequence of an abnormality in a single gene but rather the consequence of a sustained perturbation in a complex network of molecular interactions. Therefore, identifying the perturbed sub-network in a specific disease process will provide opportunities for improved diagnoses and more effective therapies. We speculate that in the near future the integration of omics data from a patient, including genome sequence and transcriptome analysis, together with an accurate TRN of heart development will provide a strong basis for indicating the status network perturbations, and greatly assist the diagnosis and the formation of a personalized therapeutic strategy for CHD. Defined disease modules can be applied as network-based biomarkers for diagnosis. A recent example illustrates this potential with the identification of network-based biomarkers for classifying breast cancer metastasis [37]. Network-based medicine is a promising concept for innovative diagnostic approaches and therapeutic strategies [38].

## Materials and Methods

### Microarray dataset

Public domain microarray data used in this study covered important temporal phases of cardiogenesis for elucidating the transcriptional landscapes active during mouse cardiac development. Array descriptions (15711 selected gene features) are in **Table S10**. We used the Robust Multi-array Average (RMA) algorithm to normalize the 239 microarrays, which were experiments all performed on the Affymetrix Mouse Genome 430 2.0 Array platform (GEO accession: GPL1261) [39].

### Comprehensive clustering algorithm

The algorithm of comprehensive clustering: *N* was defined as the number of the newly generated candidate modules in one iteration of the algorithm such that *N* = *N*_0_: *u*: *N_max_*, where N was the number of modules (*N* = *k*) to form using *k*-means clustering, *N_0_* was the initial (smallest) number of modules to start with, and u was the size of an incremental increase per iteration, up to *N_max_*. The distance measure for *k*-means clustering was 1-*r*, which was one minus the correlation of the expression profiles between genes. The expression profiles of genes in the module were centered and normalized to calculate the component-wise mean of them as the centroid. *N* was increased incrementally by u to *N_max_*, or until a single-gene module occurs (natural stopping condition). The cumulative set of co-expression gene modules for a given N was defined as the aggregation of co-expression gene modules from k-means clustering for *k* = *N* together with the cumulative set of co-expression gene modules formed for (*N*-*u*) that contained all the co-expression gene modules from multiple rounds of *k*-means clustering for *k* = *N_0_* to *k* = (*N*-*u*) with the incremental increase of *u* per round. Thus after several iterations, we obtained several cumulative sets of co-expression gene modules and each set was labeled with its corresponding *N*. Generally, co-expression gene modules newly retrieved in the iteration with larger k of clustering are more robust. We defined robustness score of each such module in a cumulative set by the probability of its existence in the iterations of *k*-means clustering that contribute to this cumulative set. ‘Existence’ is based on the criterion that a module's genes are all represented together in a module, although this single module may have other members. The more frequently the genes of a module all appear together in other modules, the higher its robustness score will be. We then sorted all the modular robustness scores in the cumulative set labeled by its specific N to retrieve the top co-expression gene modules by ordering robustness scores from large to small until we obtain complete coverage of all genes on the array. These ordered modules are collectively called the ‘top set’ of co-expression gene modules for the given N. We thus obtained several top sets corresponding to the cumulative sets. In order to identify the optimal configuration of co-expression gene modules among those several top sets, we examined each set with the DB (Davies-Bouldin) Index to calculate the overall goodness of the overlapping modular configuration in this set (where the top set of co-expression gene modules satisfied the overlap condition). The optimal set of co-expression gene modules among the top sets was determined by identifying the top set with the minimal DB Index, which indicated the optimal configuration of modules in the set. 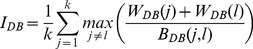
(1)


**Figure 11:**
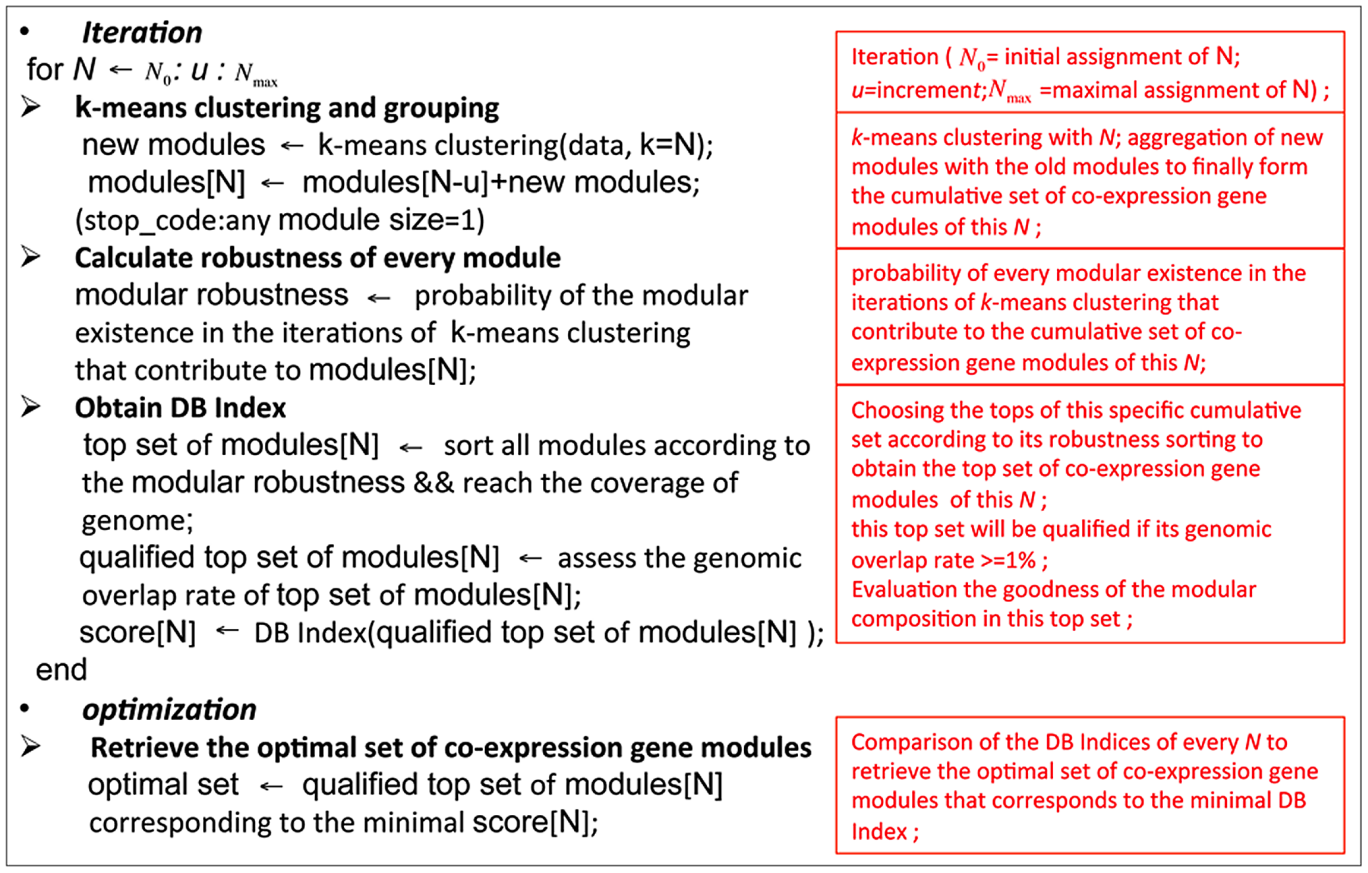
Algorithm for comprehensive clustering.

DB Index: k is the total number of modules; *W_DB_*(*j* or *l*) is the intra-module compactness for module j or module l, respectively; and *B_DB_*(*j*, *l*) is the separation between module j and module l (**Figure 11**).

A gene's transcription is regulated by a combinatorial group of TFs [32]. Each TF group can regulate several genes as demonstrated by co-expression gene module members that cooperatively accomplish specific biological functions [18]. Each of hundreds of TFs has several co-association TF subsets (size of the subset generally ranges from 2 to 5) based on genomic binding overlaps from ChIP-seq experiments [40]. Therefore hundreds (or greater) of TF combinatorial groups are possible in a given physiological/pathological process. Accordingly, hundreds of their corresponding co-expression gene modules are possible. We therefore assigned *N_0_* with 100 as the initial smallest value to consider for *k*-means clustering. Starting with the obtained modules from the *k*-means clustering of *k* and from the *k*-means clustering of (*k*+10), every gene was assigned into two modules, one module from the clustering of k and the other module from the clustering of (*k*+10), and between the two modules we found that on average (k was assigned incrementally) genes grouped together with at least one of the same gene neighbors in 80% of the total tested genes. This demonstrated good robustness of modular membership of genes between the clustering and the subsequent clustering when the increment u was set to 10. We then set the increment u to 10 for subsequent iterations of the comprehensive clustering algorithm. As we performed comprehensive clustering with overlap [18], we specified the top set of co-expression gene modules with overlap rate > = 1% as the qualified set.

### Analysis of the differential modular expression throughout developmental phases

Samples were assigned to phases as described in **Table S10**. We used ANOVA to test the differential expression in the module among the phases. The False Discovery Rate (FDR) was applied for multiple test correction. To test the differential expression in the module between one phase and the others, we applied t-tests to modular expression data and FDR was applied for multiple test correction.

### Enrichment tests

In order to identify the TFs that drive co-expression in a defined gene module, we inspected the known TFBS motifs on the gene promoters of this module. However, matching hits of known TFBS motifs on gene promoters can be spurious false positive hits. We evaluated the matching hits of TFBS motifs in a module against a hypergeometric distribution to exclude the cases of random motif matches in the module. The hypergeometric function describes the probability of exactly k successes in n draws from a finite population size T where the number of successes in the whole population is m. 
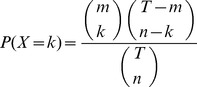
(2)


By letting k =  integers k through n, the hypergeometric probabilities may be summed to find P(*X*> = *k*), and obtain the probability of the proposition that the number of successes observed or a higher number of successes in n draws would occur by chance. If this is less than 0.05 (a typical choice), we demonstrate that the situation of k successes in n draws is significantly unlikely due to chance. Thus, the attribute corresponding to the success is defined to be overrepresented in the n draws against the background of m successes in T population. In the case of evaluating the hits of the known TFBS motif onto the gene promoters in a module, this approach is specified as the TFBS motif enrichment test. In our study, for any known TFBS motif, k was the number of gene promoters that had the binding site of this TFBS motif in the co-expression gene module being tested, n was the number of genes in this module and m was the number of gene promoters that had the binding site of this TFBS motif in the tested genome and T was the number of genes in the tested genome. Via referring the overrepresented/enriched TFBS motifs to their corresponding TFs, we inferred their transcriptional relations in each module.

The hypergeometric test was also used for the pathway enrichment tests in this study. For any pathway, k was the number of genes or gene products which had functional positions in a given pathway from the co-expression gene module being tested, n was the number of genes in this module, m was the number of genes or gene products that had functional positions in the complete pathway and T was the number of genes in the tested genome.

Overrepresentation was assessed when *k*>1 for a given TFBS motif or pathway.

### Databases

MPromDb (Mammalian Promoter Database) is a curated database that annotates gene promoters identified with ChIP-seq which is one of the most robust approaches of defining gene promoters [41]. The ChIP-seq data sets of RNAP-II and various TFs were included in significance tests to retrieve the gene promoters archived in this database. To complete retrieving the gene promoters in the genomic scope, gene promoters (from 5000 bp upstream the TSS to 1000 bp downstream the TSS) that are not covered by MPromDb were retrieved from UCSC genome browser [42].

JASPAR is a curated database of known TFBS motifs for various organisms from the experiment-based literature [31], and was used in this study to identify motif matches in the promoters of module genes. TFBS motifs are stored as a position weighted matrix (PWM) in JASPAR.

TRANSFAC is a curated database of eukaryotic transcription factors, and includes experimentally-proven binding sites and regulated gene targets [11], [12]. Publicly available TRANSFAC knowledge was summarized in **Figure S1**.

Wikipathways is an open, collaborative platform dedicated to the curation of biological pathways, in order to facilitate the contribution and maintenance of the pathway information [29], [30]. The Wikipathways knowledgebase was used in this study as the basis for evaluating pathway overrepresentation in the defined co-expression modules.

We collected the ChIP-seq data for the sampled TFs of mouse from Encode database (Experiment details for the sampled TFs can be found in **Table S11**). ChIP-seq data reveal genomic binding sites for TFs. The regulated gene pool of every sampled TF was estimated from an analysis of proximity between genomic binding sites from ChIP-seq and the gene promoters.

### TFBS motif alignment

TFBS is a motif alignment program that was obtained from JASPAR (http://tfbs.genereg.net/). It is able to align the TFBS motif onto the gene promoters either in the co-expression gene module or in the genome. In this program, within one promoter sequence, the PWM of one TFBS motif slides over the sequence in 1-bp increment, and each potential binding site is evaluated against the PWM on both strands through a quantitative score. The quantitative score for a potential binding site of a specific TFBS motif is produced by summing the relevant nucleotide PWM values, analogous to the probability of observing this potential binding site given the source of this TFBS motif [43]. Then we defined the hits of the TFBS motif on the promoter with the quantitative scores above the corresponding threshold as the predicted binding sites that contribute to k or m in the TFBS motif enrichment test.

### Data access

Results are available in the Supporting Tables. In-house software used to conduct this investigation is available upon request. The normalized expression dataset is available in a compressed archive (Dataset S1).

## Supporting Information

Dataset S1 **Scaled and normalized microarray expression data used in this study.**
(ZIP)

Figure S1 **Literature-based mouse transcriptional interactions from the public version of the TRANSFAC database.**
(TIFF)

Table S1 **Genes associated with congenital heart disease.**
(XLS)

Table S2 **Optimal modules identified in this study.** Member Entrez Gene IDs are listed for each of the 765 gene modules identified.
(XLSX)

Table S3 **Assignment of development phase to modules according to expression levels.** This table also contains adjusted p-values (FDR<.05) for ANOVA and individual t-tests of differential expression.
(XLSX)

Table S4 **Module counts of enriched pathways from Wikipathways.**
(XLSX)

Table S5 **Module counts of enriched TFBS in the promotors of module gene members.** Enrichments were assessed for promoters using TFBS motifs for mouse, human and rat from the JASPAR database.
(XLSX)

Table S6 **Cross table of module counts for TFBS- and pathway-enrichments.**
(XLSX)

Table S7 **Inferred transcription factor-target relationships.** This table contains a complete list of module genes and respective enrichments for TFBS. The column ‘TFBS Alignment on the Promoter of Gene Target’ contains a ‘1’ if the gene has an alignment with the enriched transcription factor binding site motif for the respective transcription factor, and a ‘0’ if that gene did not have an alignment with the enriched motif.
(XLSX)

Table S8 **Inferred gene co-regulations by two transcription factors.**
(XLSX)

Table S9 **Inferred gene co-regulations by three transcription factors.**
(XLSX)

Table S10 **Listing of expression microarray CEL files used in this study, and annotation of the developmental stages of their respective samples.**
(XLSX)

Table S11 **Listing of ChIP-seq source data used in this study.**
(XLSX)
