# Supplementary figures and images for: A Provisional Gene Regulatory Atlas for Mouse Heart Development
Source: PLoS One. 2014 Jan 8;9(1):e83364. doi: 10.1371/journal.pone.0083364 (PMC3885437; doi:10.1371/journal.pone.0083364)

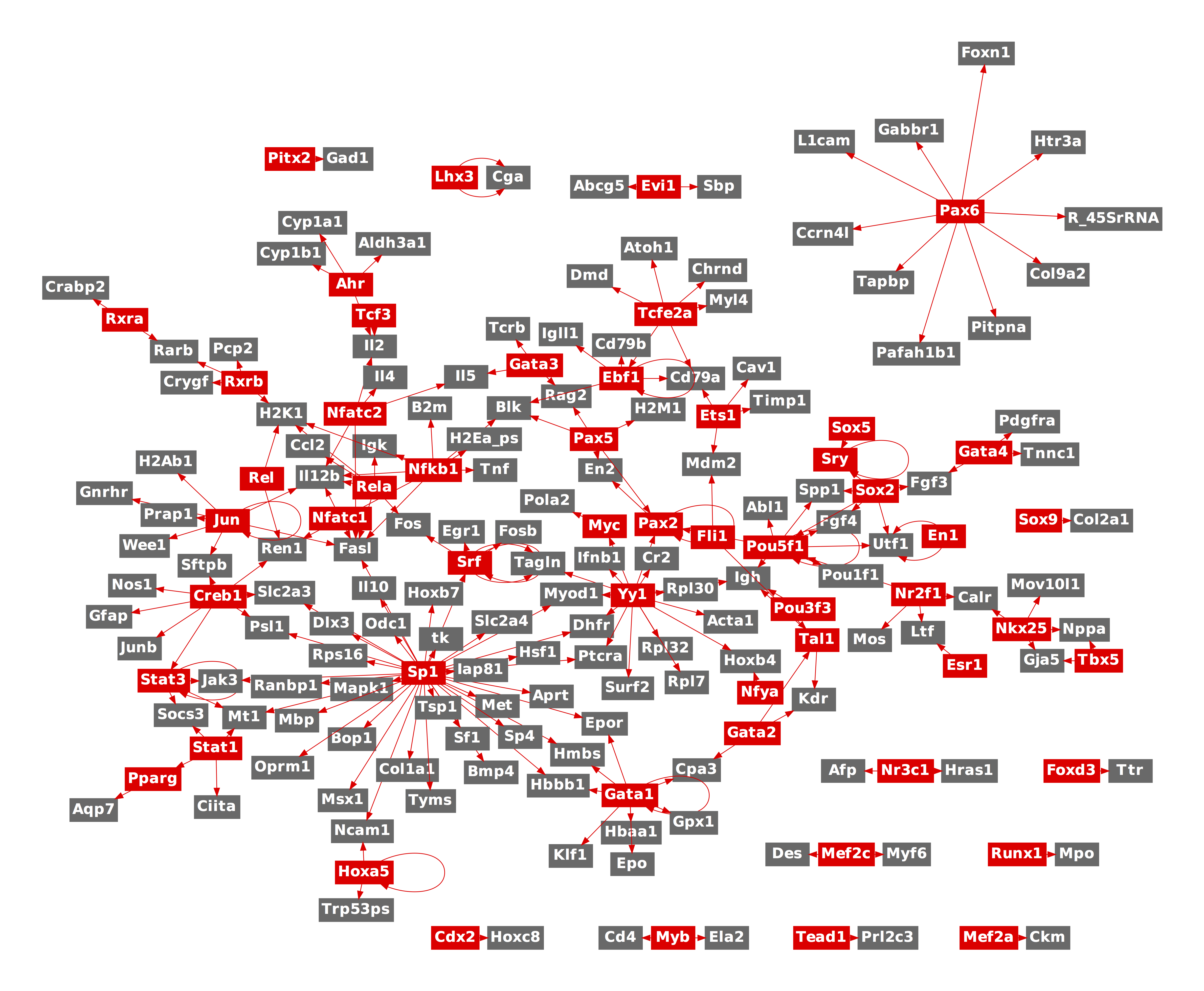

Supplement: Figure S1 — Literature-based mouse transcriptional interactions from the public version of the TRANSFAC database. (TIFF) [file pone.0083364.s002.tiff]
